# Secondary infections in critically ill patients with COVID-19 receiving steroid therapy

**DOI:** 10.1177/00368504231207209

**Published:** 2023-10-30

**Authors:** Alex K Pearce, Qais Zawaydeh, W Cameron McGuire, Abdurrahman Husain, Claudia Ayoub, Daniel A Sweeney, Shannon A Cotton, Atul Malhotra

**Affiliations:** 1 Division of Pulmonary, Critical Care, Sleep Medicine and Physiology, 315531 University of California, San Diego, La Jolla, CA, USA; 2 Department of Internal Medicine, 541618 Eisenhower Health, Palm Springs, CA, USA; 3 Division of Pulmonary Critical Care Medicine, 2655 Texas A&M School of Medicine, Baylor University Medical Center, Dallas, TX, USA; 4 Department of Internal Medicine and Pediatrics, 21693 University of Mississippi Medical Center, Jackson, MS, USA

**Keywords:** Intensive care unit, steroids, coronavirus disease 2019, secondary infection, critical care outcomes

## Abstract

Secondary infections can occur during or after the treatment of an initial infection. Glucocorticoids may decrease mortality in patients with severe COVID-19; however, risk of secondary infection is not well described. Our primary objective was to investigate the risk of secondary infection among critically ill patients with COVID-19 treated with glucocorticoids. We examined patients with COVID-19 being treated in the intensive care unit at two academic medical centers from 1 to 7/2020. One hundred-seven patients were included. Of these, 31 received steroids and 76 patients did not. Analysis of the larger cohort was performed followed by a matched pairs analysis of 22 steroid and 22 non-steroid patients. Secondary infection was seen in 14 patients (45.2%) receiving steroids compared to 35(46.1%) not receiving steroids (p = 0.968). Secondary infections were most frequently encountered in the respiratory tract. Escherichia coli and Staphylococcus aureus were the most frequently identified organisms. Mortality was 16.1% in the steroid-treated group compared to 23.7% in the control group (p = 0.388). After performing matched pairs analysis and multivariable logistic regression there was no significant difference between secondary infection or mortality and steroid receipt. Secondary infections were common among critically ill patients with COVID-19, but the incidence of secondary infection was not significantly impacted by steroid treatment.

## Introduction

The COVID-19 pandemic has led to devastating consequences for afflicted patients, communities, and healthcare infrastructures. One clinical manifestation is the development of acute respiratory distress syndrome (ARDS) from severe COVID-19.^1^ Many different types of pharmacological therapies have been trialed in the treatment of COVID-19 with mixed success.^2^ The landmark Dexamethasone in Hospitalized Patients with COVID-19 (RECOVERY trial) indicated a significant mortality reduction in critically ill patients hospitalized with COVID-19 treated with dexamethasone compared to placebo.^3^ Subsequent real-world cohort studies have replicated the positive impact of steroids in severe COVID-19.^4^ Although evidence points to steroids being beneficial in the treatment of COVID-19 patients requiring respiratory support, the risks, benefits, and optimal doses are not fully characterized.^5^ Prior reports from observational studies have suggested that steroids may have deleterious effects on viral illnesses such as influenza.^6,7^ There are also clearly difficulties in assessing the efficacy of steroids in retrospective studies given that the patients with the highest perceived mortality risk are disproportionately treated with steroids. This so-called “confounding by indication” may suggest underlying disease for which steroids are given contributes to poor outcomes rather than the steroids per se.

Secondary infections can occur during or after the treatment of an initial infection. Given the immunomodulatory effects of steroids, there is concern that the use of glucocorticoids may increase the risk of secondary infections. Based on reports of fungal secondary infections in COVID-19 patients, we examined patients prior to the RECOVERY publication to assess the potential impact of glucocorticoid use.^8–11^

Two recent meta-analyses of randomized trials found consistent benefits of steroids in COVID-19 without any major adverse effects.^5,12^ However, the reports acknowledged uncertainty about adverse event rates due to variable definitions and inconsistent reporting of serious adverse effects across studies. The Adaptive COVID-19 Treatment Trial-4 (ACTT-4) comparing Baricitinib versus dexamethasone for adults hospitalized with COVID-19 showed no increase in new infections in the steroid-treated group.^13^ However, other reports of secondary infections including bacteremia, ventilator-associated pneumonia (VAP) and pulmonary aspergillosis in patients with COVID-19 receiving corticosteroids indicate additional studies of adverse events are warranted.^14,15^

Identifying this gap in the literature, we sought to characterize the relationship between steroid use and secondary infections in patients with COVID-19. Specifically, we tested the hypothesis that steroids may increase the risk of secondary infections among critically ill patients with COVID-19. In addition, we sought to investigate confounding by indication by examining the outcomes of patients who received steroids following recognized secondary infection.

## Methods

This was a retrospective cohort study of 129 consecutive patients with COVID-19 admitted to the ICU at two large, tertiary care, academic medical centers between January 2020 and July 2020. The enrollment time interval was selected because it preceded the release of the RECOVERY trial in *NEJM*, after which dexamethasone therapy became the standard of care in critically ill patients with COVID-19.^3,16^ Patient confidentiality was protected using de-identified numbers and the study was approved by the UCSD institutional review board (Study title: COVID Registry and Clinical Data Repository, IRB #200498, approval date 3/31/2020). Consent was waived by the IRB. Procedures were followed in accordance with the ethical standards of the responsible committee on human experimentation (institutional or regional) and with the Helsinki Declaration of 1975. Inclusion criteria consisted of 1) Patients who tested positive for COVID-19 by nasopharyngeal swab using Nucleic Acid Amplification Test/Reverse Transcription-Polymerase Chain Reaction (NAAT/RT-PCR) techniques according to the Centers for Disease Control and Prevention (CDC) guidelines^17^ and 2) critically ill patients defined by meeting clinical criteria for admission to the ICU. There were no specific exclusion criteria.

## Data collection

Data on 129 patients were retrieved from the electronic medical record manually by trained physicians. These data included: demographics (i.e. gender, age), body mass index (BMI), average daily steroid dose (in prednisone equivalents), hospital day at which the steroid was administered, hospital day at which secondary infection was detected, the total number of positive cultures (blood, sputum, and/or urine), use of antibiotics at the time of, or in response to, a positive culture, positive *Aspergillus* galactomannan (blood or bronchoalveolar lavage sample), sequential organ failure assessment (SOFA) score^18^ on ICU day 0, and 28-day mortality. Microbiologic data documenting specific organisms were also reported as descriptive data although single blood cultures with common contaminant organisms were not included. Culture collection was driven based on the clinical course of the patient or suspicion of infection.

To assess better the risk of secondary infections associated with steroids, and to account for confounding by indication, only patients who received steroids prior to the first positive culture were included in the steroid group for the primary analysis. If the timing of steroid and secondary infection was unclear by chart review the case was excluded. If patients received steroids after a positive culture, they were analyzed separately as an exploratory analysis to assess confounding by indication of mortality.

In total, we analyzed the 107 patients who met our criteria for the primary analysis. To adjust for the impact of age group and severity of illness (assessed by SOFA score) on outcomes, for a subset, we utilized a matched pairs design to compare secondary infection and mortality in patients treated with and without steroids. In addition, we performed multivariable logistic regression as another method to assess the relationship between the use of steroids and the incidence of secondary infection and mortality in the larger cohort. The remaining 19 patients who received steroids after documentation of secondary infection were analyzed separately out of interest for potential confounding by indication.

## Outcome measures

The primary outcome was secondary infection defined by a new positive blood, sputum, and/or urine culture requiring treatment with antibiotics. The secondary outcome was 28-day mortality from day zero of admission to the ICU. The explanatory variable of interest was glucocorticoid therapy. For matched pairs analysis, we defined *a priori* important covariates including SOFA score and age to allow matched comparisons between steroid and non-steroid-treated groups. For the larger cohort, logistic regression, both adjusted and unadjusted odds ratios were calculated. In the adjusted model age, sex, BMI, and SOFA score were selected as covariates based on *a priori* criteria. The Hosmer-Lemeshow test was used to assess for goodness of fit. We compared dichotomous variables using a chi-squared test and for non-normally distributed data we utilized non-parametric tests. P-values <0.05 were considered statistically significant. Statistical analyses were performed using IBM SPSS Statistics for Mac OS (version 28, SPSS IBM Corp., Armonk NY, USA).

## Results

Out of the 107 patients included in the final analysis, we identified 31 who received steroids and 76 who did not (baseline and demographic characteristics in Table 1). In the steroid-receiving cohort average age was 49 years (SD 16), 51.6% were female, and average BMI was 33.2 kg/m2 (SD 8.0). In the group who did not receive steroids average age was 61 years (SD 13.6), 30.3% were female, and average BMI was 30.7 kg/m^2^ (SD 6.8). In this cohort, patients not treated with steroids had a 46.1% rate of secondary infection compared to 45.2% of patients treated with steroids (p = 0.968) (Table 2). Mortality was 16.1% in the steroid-treated group compared to 23.7% in the control group (p = 0.388) (Table 2). After performing multivariable logistic regression accounting for age, sex, SOFA score and BMI, steroid use was not associated with increased risk of secondary infection (p = 0.679). Steroid use also was not associated with increased survival in this analysis (p = 0.892). Applying the matched pairs design resulted in 22 patients from the steroid group being compared to 22 patients not receiving steroids (N = 44). After matching patients not treated with steroids, 36.4% had a documented secondary infection compared to 50.0% of patients treated with steroids (p = 0.361) (Table 2). Mortality was 18.2% in the steroid-treated group compared to 13.6% in the matched nonsteroid-receiving group (p = 0.680) (Table 2).

**Table 1.** Baseline characteristics of study participants.

Variable	Steroids (n = 31)	No steroids (n = 76)
Age, years (SD)	48.8 (16.1)	60.8 (13.6)
Sex (%, n)		
Female	51.6 (16)	30.3 (23)
BMI (SD)	33.2 (8.0)	30.7 (6.8)
Comorbidities (%)		
Chronic pulmonary disease	9.7	6.6
Hypertension	29.0	35.5
Chronic kidney disease	12.9	7.9
Diabetes mellitus	25.8	25.0

**Table 2.** Secondary infection and mortality in non-steroid vs steroid receiving patients.

Outcomes	(-) steroids	+ steroids	P-value
Entire Cohort (n = 107)	n = 76	n = 31	
Secondary Infection (%, n)	46.1 (35)	45.2 (14)	0.968
Mortality (%, n)	23.7 (18)	16.1 (5)	0.388
Matched Cohort* (n = 44)	n = 22	n = 22	
Secondary Infection (%, n)	36.4 (8)	50.0 (11)	0.361
Mortality (%, n)	13.6 (3)	18.2 (4)	0.680

In patients receiving steroids, there was no significant difference in the average daily steroid dose (converted to oral prednisone equivalents) in patients who did not develop secondary infection compared to patients who did develop secondary infection [44.8 mg (SD 24.7) vs 61.2 mg (SD 37), U = 153, p = 0.186].

Of patients with positive microbiologic data including urine, blood, or respiratory cultures, *Escherichia coli* and *Staphylococcus aureus* were the most frequently isolated causative organisms. The respiratory site was the most common source of secondary infection (43.4%). No *Aspergillus* species were identified on culture and no positive galactomannan tests were reported for the patients enrolled. Cultures were obtained on average 7 days (SD 7) into the hospitalization. Additional detailed microbiologic data including microorganisms and infection source are summarized in Table 3.

**Table 3.** Distribution of microorganisms and sources of infection.

	Blood	Respiratory	Urine	# of positive cultures n (%)	Hospital Day positive culture collected (mean ± SD)
Source of infection n (%)	38 (27.9%)	59(43.4%)	39 (28.7%)	Total 136	
Organism					
Escherichia coli	4	7 (4 ESBL)^a^	15 (7 ESBL)^a^	26 (19.1)	9 ± 10
Enterococcus spp.	4	1	10 (1 VRE)^b^	15 (11.0)	8 ± 8
Enterobacter spp.	3	9	1	13 (9.6)	14 ± 9
Klebsiella pneumoniae	2	5	2	9 (6.6)	7 ± 8)
Staphylococcus aureus	10 (2 MRSA)^c^	14 (2 MRSA)^c^		24 (17.7)	8 ± 9
Pseudomonas aeruginosa	6	6	4	16 (11.8)	9 ± 7
Citrobacter spp.		4	2	6 (4.4)	16 ± 13
Streptococcus pneumoniae		2		2 (1.5)	0
Proteus mirabilis	1	2	5	8 (5.9)	11 ± 13
Candida spp	3			3 (2.2)	20 ± 5
Stenotrophomonas spp	1	3		4 (2.9)	20 ± 29
Coccidioides immitis		1		1 (0.4)	
Other	4	5		9 (6.6)	

Notes. Values are expressed as number and percentage of total positive cultures. Please note that several patients had multiple sources of infection, and several grew more than 1 organism. Microbiology of the positive cultures that were obtained in the 107 COVID-19 patients. For cultures with timing data available, the mean hospital day of positive culture collection is reported. Spp Species. ^a^ESBL- Extend spectrum beta-lactamase ^b^VRE- Vancomycin-resistant enterococcus ^c^MRSA- Methicillin-resistant Staphylococcus aureus.

Of the group who received steroids after documentation of secondary (n = 19) infection, mortality was 57.9% compared to the group who received steroids prior to secondary infection (n = 31) where mortality was only 16.1% (p = 0.004). The group who received steroids after secondary infection compared to the group who received steroids before secondary infection was significantly older (60 ± 12 vs 49 ± 16 years, p = 0.009) and mostly male (84% vs 45% male, p = 0.02). SOFA scores and comorbid conditions (DM, CKD, HTN, lung disease) were not significantly different between groups.

## Discussion

Our findings are important for several reasons. First, we have conducted a comparison of patients with COVID-19 who did and did not receive steroids and observed no increase in secondary infection associated with steroid administration. At baseline, patients who received steroids were younger and more likely to be female; however, after adjusting for age and gender via statistical analyses there was no difference in secondary infection between groups. On further analysis, matching major covariates including SOFA score as a marker of severity of illness and age we again found no difference between steroid use and secondary infection. Additionally, among the patients who received steroids (n = 31) we observed only three deaths in the group who developed secondary infection and two deaths in patients who did not develop secondary infection. We are reassured by this finding as it suggests that the development of secondary infection after receipt of steroids is not independently associated with increased mortality. Our data are helpful particularly when put into the context of the existing literature. The RECOVERY trial showed benefits to dexamethasone for critically ill patients with COVID-19 but did not report data regarding the incidence of secondary infection.^3^ Other data regarding a risk of secondary infection and steroid use in patients with COVID-19 use are mixed.^14,19^ Thus, we and others have been concerned about the possibility of steroid-induced superinfection in vulnerable patients.^14,15^ Our data add to the existing literature by providing some assurance that steroids do not increase risk of secondary infection in critically ill patients with COVID-19.

Second, we provide data describing the microbiologic prevalence of organisms contributing to secondary infection and specifically looked for *Aspergillus* infection in our cohort due to concerns about steroid-induced susceptibility. We did not find any evidence of *Aspergillus* infection in our patient cohort which differs from other studies that reported rates of invasive pulmonary aspergillosis infection of 20–30% in critically ill patients with COVID-19.^20,21^

Third, questions remain regarding the safety, tolerability, optimal dosing/timing,^22,23^ and type of steroid^24^ to be used. Currently, in the acute setting, hospitalized patients requiring supplemental oxygen, dexamethasone 6 mg daily for 10 days is recommended by the National Institute of Health COVID-19 treatment guidelines.^16^ In our cohort, glucocorticoid use and adjusted daily dose of glucocorticoid were not independently associated with an increased risk of secondary infection. Our data may provide some guidance in the design of future studies regarding optimal steroid dosing.^25,26^

Some observational studies have suggested the potential deleterious effects of steroids on viral illness. For example, steroids have been associated with worse outcomes in influenza pneumonia compared to patients who do not receive steroids.^27,28^ The concern with such studies is due to ‘confounding by indication’ whereby sicker patients are given steroids (for example steroids given in the setting of shock) and thus poor outcomes may be a function of severity of illness rather than steroids per se. To our knowledge, no large, randomized trials have shown poor outcomes with steroid therapy in influenza infection. Our study design helps to mitigate the concern about confounding by indication since we analyzed separately patients who received steroids after established secondary infection (‘late’ steroids). Mortality in the “late” steroid group was 57.9% (considerably higher than our study participants), emphasizing the importance of addressing confounding by indication and the need for randomized trials to draw rigorous conclusions. In addition, mortality was not significantly different between our steroid and non-steroid groups, providing reassurance regarding steroid therapy in COVID-19.

## Limitations

Despite our study's strengths, we acknowledge some limitations. First, although our cohort of 107 patients is reasonable, we had only a modest sample size for comparison after matching and the sizes of the steroid-treated subgroups were small. Thus, we may have had limited power to make definitive conclusions, but believe our findings still provide some reassurance regarding the use of steroids in COVID-19. Second, we did not conduct a randomized trial, so the possibility exists for residual confounding. We did our best to control for known covariates such as age and SOFA score as markers of illness severity. Nonetheless, we are highly supportive of further randomized trials to assess the optimal use and associated risk of steroids in critically ill patients. Third, given the retrospective nature of our design, we relied on clinicians to obtain culture data whenever clinically indicated. We cannot exclude whether sub-clinical infections were occurring or indeed whether important superinfections were missed based on our design. Also, we do not have details regarding central line, Foley catheter usage, or duration of mechanical ventilation which may influence the risk for secondary infection. Some centers have suggested the use of surveillance bronchoscopy prior to initiating steroids^29^ although this practice requires further study but may yield more positive (including false positive) culture results. Despite these limitations, we believe our study has several strengths and hope that it encourages further research in this important area.

## Conclusions

Despite considerable research, the therapeutic options for COVID-19 remain limited. Our data provide some confidence that the use of glucocorticoids in the treatment of these patients is likely safe. Although we are highly supportive of larger well-controlled studies, our findings may suggest that steroids are unlikely to predispose to secondary infections. Fungal superinfection does not appear to be a major concern based on our findings.
